# Draft genome of *Pasteurella oralis* WCHPO000540 recovered from an animal bite wound

**DOI:** 10.1128/mra.00613-25

**Published:** 2025-07-23

**Authors:** Linwan Zhang, Yu Feng, Zhiyong Zong

**Affiliations:** 1 Center of Infectious Diseases, West China Hospital, Sichuan University 12530, Chengdu, China; 2 Department of Clinical Research Management, West China Hospital, Sichuan University 12530, Chengdu, China; 3 Laboratory of Pathogen Research, West China Hospital, Sichuan University 12530, Chengdu, China; 4 Division of Infectious Diseases, State Key Laboratory of Biotherapy, Chengdu, China; Rochester Institute of Technology, Rochester, New York, USA

**Keywords:** *Pasteurella oralis*, wound secretion, animal bite

## Abstract

We report the draft genome sequence of *Pasteurella oralis* isolated from animal bite wound secretion. The assembly comprises 30 contigs totaling 2,388,559 bp, with an N*50* of 348,256 bp and a GC content of 37.15%.

## ANNOUNCEMENT

Bite-wound infections typically arise from polymicrobial aerobic–anaerobic communities and frequently involve zoonotic pathogens not covered by standard empiric therapies. In a multicenter survey, *Pasteurella* spp. were recovered from over 50% of companion animal bite wounds (1). *Pasteurella oralis* (formerly “species B”) is a Gram-negative, non-motile coccobacillus that, like other members of the genus, forms small, smooth, non-hemolytic colonies on blood agar; it colonizes the oral mucosa of domestic and wild mammals and can cause severe soft-tissue infections (2, 3). To date, only two *P. oralis* genomes have been deposited in GenBank, with one derived from metagenome origin, constraining our understanding of its genetic diversity and virulence potential. Here, we present another genome, assembled from pure culture, aiming to enable more accurate molecular diagnostics and therapeutic strategies for managing bite wound infections. This retrospective genome study has been approved by the Ethical Committee of West China Hospital, with informed consent being waived.

In 2017, a strain designated WCHPO000540 was isolated from the secretion of a bite wound by an unidentified *Mustelidae* animal from a patient at West China Hospital. The sample was first cultured on Columbia blood agar at 37°C aerobically for 24 h. A single colony was obtained by three rounds of streaking and then cultured in Luria-Bertani broth at 37°C overnight for proliferation. Genomic DNA was extracted with the QIAamp DNA Blood Mini Kit (Qiagen, Hilden, Germany), and a 350-bp insert library was prepared using the NEBNext Ultra II kit (New England Biolabs, Ipswich, MA). Sequencing on the Illumina HiSeq X10 platform (Illumina, San Diego, CA) generated 3,035,812 paired-end reads (150 bp) totaling 0.91 Gb (381× coverage). For all downstream bioinformatic pipelines, default parameters were applied unless otherwise noted. Raw reads were quality-trimmed with Trimmomatic v0.39 (4) and subsampled to 200× coverage using SeqKit v2.8.2 (5). We assembled the genome with SPAdes v3.11.0 (6) under careful mode with error correction, followed by species identification using FastANI v1.34 (7) against *P. oralis* type strain CCM 7950^T^ (accession no. JBHUFP000000000), which yielded an average nucleotide identity (ANI) of 98.25%, confirming WCHPO000540 as *P. oralis*. We summarized assembly metrics with QUAST v5.3 (8) and assessed genome quality using CheckM2 v1.1.0 (9). Annotation was performed via the NCBI Prokaryotic Genome Annotation Pipeline v6.10 (10). Compared with the type strain CCM 7950^T^ (Table 1), the genome demonstrated comparable assembly metrics and gene counts. They each assemble into roughly 30 contigs totaling about 2.4 Mb, share a GC content of approximately 37.2% and have N*50* values in the 350–400 kb range. Meanwhile, antimicrobial susceptibility testing following the Kirby–Bauer disk diffusion method per CLSI guidelines was performed, demonstrating that the strain was susceptible to chloramphenicol (≤34 mm), azithromycin (≤23 mm), ceftriaxone (≤34 mm), erythromycin (≤18 mm), levofloxacin (≤32 mm), tetracycline (≤30 mm), and penicillin G (≤23 mm).

**TABLE 1:** Assembly and annotation statistics between *Pasteurella oralis* type strain CCM 7950^T^ and strain WCHPO000540

	WCHPO000540	CCM 7950^T^
Assembly statistics		
Contigs	30	27
Total length (bp)	2,388,559	2,446,620
GC (%)	37.15	37.32
N50 (bp)	348,256	400,104
Coverage	251.18×	218×
Annotation statistics		
Genes (total)	2,272	2,347
CDSs (total)	2,214	2,289
Genes (coding)	2,175	2,252
CDSs (with protein)	2,175	2,252
Genes (RNA)	58	58
rRNAs (5S, 16S, 23S)	2, 1, 1	3, 1, 1
tRNAs	50	49
ncRNAs	4	4
Pseudo Ggenes (total)	39	37
CDSs (without protein)	39	37
CRISPR Arrays	3	2

## Data Availability

The draft genome assembly of *P. oralis* WCHPO000540 is available in GenBank under accession number NXGB00000000. Raw sequence reads have been deposited in the Sequence Read Archive (SRA) under accession number SRR7031255.
